# Water Recovery from Laundry Wastewater by Integrated Purification Systems

**DOI:** 10.3390/membranes15040125

**Published:** 2025-04-14

**Authors:** Aleksandra Klimonda, Izabela Kowalska

**Affiliations:** Faculty of Environmental Engineering, Wroclaw University of Science and Technology, Wybrzeże S. Wyspiańskiego 27, 50-370 Wroclaw, Poland

**Keywords:** greywater, circular economy, pressure-driven membrane process, cationic surfactants, adsorption, ion exchange, esterquat

## Abstract

Integrated systems for water recovery from laundry wastewater were investigated to close the water cycle loop. The grey water from the washing cycle of cotton fabrics with a softening detergent was prefiltered, purified in a low-pressure membrane process, and further purified in a high-pressure membrane process or alternatively in an adsorption process. In all of the proposed systems, the recovered water was clarified and completely free of cationic surfactants. The system based on nanofiltration as the final stage of purification allows soft water, which promotes better cleaning results, reduces the consumption of detergents, and extends the lifetime of the devices involved. Adsorption on activated carbon effectively reduced the concentration of organic compounds, including fragrance compounds.

## 1. Introduction

The laundry business is widely recognised as a significant water consumer. On average, one hotel room generates 6.4 kg of laundry per day [1] while washing 1 kg of textiles requires approximately 15 L of fresh water [2] and generates wastewater containing a variety of organic and inorganic pollutants. Organic contaminants include soaps, detergents, fabric softeners, and grease; while inorganic pollutants consist of heavy metals, sand, silt, clay, metal ions, particles, phosphorus, and nitrogen compounds [3,4].

In the initial stage of the laundry cycle, the main impurities (fats, oils, greases, and suspended solids) [5] are removed from the textiles using ‘heavy duty’ detergents. These detergents are primarily composed of a mixture of anionic surfactants, such as linear alkylbenzene sulfonates (LABS), sodium lauryl ether sulphate (SLES), sodium dodecyl sulphate (SDS), and ether sulphates with nonionic surfactants such as alcohol ethoxylates. Additionally, builders, enzymes, polymers, and sometimes bleaches are included as active ingredients in these formulations [6]. Anionic surfactants exhibit excellent washing properties; however, they are sensitive to the presence of multivalent ions in hard water. In contrast, nonionic surfactants are highly soluble and largely unaffected by water hardness, but they are generally less effective than anionic surfactants in removing dirt and particulate matter from fabrics [7]. During the rinse stage, impurities and residual detergents are washed out of the laundry load. In the following stage, fabric softeners are applied to enhance the softness of the fabric, provide antistatic properties, and prevent static charge on the fibre. The softening effect is mainly attributed to the action of cationic surfactants.

Surfactants are classified into four groups (anionic, nonionic, cationic, and amphoteric) based on their molecular structure and charge of the hydrophilic part. Because of the positive charge of the ionic group in cationic surfactants, these compounds exhibit the ability to adsorb onto negatively charged surfaces. This phenomenon affects the antistatic and biocidal properties of cationic surfactants; however, the best softening properties are exhibited by cationic surfactants characterised by two long alkyl chains [8].

Currently, the primary cationic surfactants used in fabric softeners are esterquats, including diethyloxyesterdimethylammonium chloride (DEEDMAC), Hamburg esterquat (HEC), and triethanolamine-based esterquat (TEAQ) [9]. Esterquats are quaternary ammonium salts containing two long fatty acid chains (comprising 16 to 18 carbon atoms) with two weak ester bonds. The first compounds in the esterquat group were patented in the 1970s. Due to their softening effect, favourable environmental profile, and lower toxicity, esterquats replaced previously used dialkyldimethylammonium salts in the 1990s [10]. In 2024, the esterquat industry was valued at USD 2.6 billion and is projected to reach more than USD 6.2 billion by 2034 [11].

The composition of laundry wastewater (LWW) can vary depending on the type of laundry facility (domestic, hospital, or industrial) due to differences in the types of fabrics washed, the detergents used and the nature of the contaminants that are removed (Table 1). For example, hospital laundry wastewater often contains blood or urine [12] as well as significant amounts of disinfecting chemicals (such as peroxides, chlorine, and aldehydes). It should be noted that the main load of pollutants is generated during the first stages of laundry, while the wastewater obtained during the finishing is generally less contaminated.

**Figure 1:**
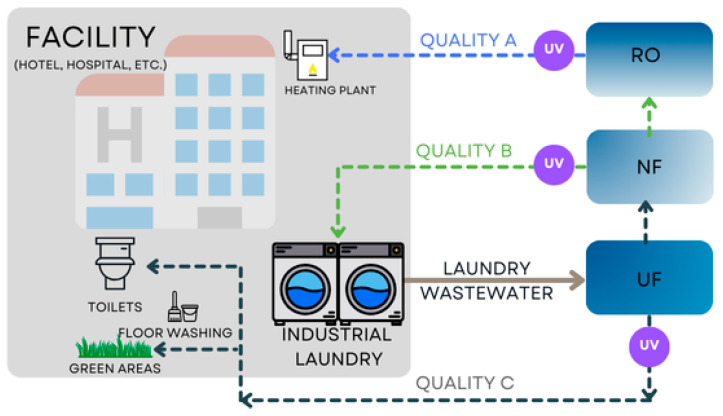
Scheme of water recycling from industrial laundries.

Industrial laundry wastewater accounts for approximately 10% of urban wastewater [19] and offers an opportunity to mitigate shortages and reduce environmental impacts [20]. Depending on the efficiency of the treatment processes and the quality of the purified water, reclaimed water can be reused for various non-potable applications (Figure 1, Table 2). Moderately treated wastewater can be used for flushing toilets and irrigation of the landscape, while advanced treatment methods, such as membrane filtration or oxidation processes, allow reuse in laundry operations [21]. In cases of high purification efficiency (e.g., reverse osmosis process), recovered water may even be suitable for boiler feed applications, enhancing industrial water sustainability.

The treatment of laundry wastewater (LWW) employs various methods aimed at minimizing its environmental footprint and enabling water reuse. These methods can be classified into physicochemical, biological, or integrated systems [22,23]. Due to its complexity and variety depending on the specification of the washing process, laundry wastewater treatment requires multiple approaches [19,23,24].

Recent studies have examined a wide range of LWW treatment technologies, including conventional physicochemical processes such as coagulation and flocculation [13,16], biological treatment [19], and advanced oxidation processes (AOPs), including ozonation and UV/H_2_O_2_ [15,21]. For example, Deressa et al. [13] reported that coagulation/flocculation at pH 6 with a coagulant dose of 2.5 g/L reduced anionic surfactants, COD, and turbidity by 92%, 83%, and 85%, respectively, in domestic LWW. In industrial applications, Nascimento et al. [16] demonstrated that combining coagulation, flocculation and sedimentation with microfiltration achieved removal efficiencies of 72% for anionic surfactants, 69% for COD, and 99% for turbidity. AOPs have demonstrated high efficiency in the degradation of organic pollutants, yet they often involve significant operational costs and energy requirements, limiting their widespread application in industrial laundries [17].

More recently, pressure-driven membrane processes (PDMPs), including ultrafiltration (UF), nanofiltration (NF), and reverse osmosis (RO), are being increasingly used in laundry wastewater treatment, both as unit processes and in integrated systems [5,16,20]. Compared to conventional technologies, PDMPs offer high pollutant removal efficiency and a compact footprint, but are limited by membrane fouling.

The application of microfiltration, ultrafiltration and nanofiltration as unit processes in LWW treatment was effective in the removal of COD and turbidity, with reported efficiencies of 57 ± 0.8% [25], 88% [26], and 97% [27] for COD, and 77 ± 2.8% [25], 98.4% [26], and 98% [27] for turbidity. PDMPs integrated into treatment systems with conventional methods may yield even better results. For example, implementing coagulation as a pretreatment step before MF has led to a 61% reduction in COD and 100% decrease in turbidity [28]. Similarly, a treatment system combining granular activated carbon adsorption followed by UF achieved 87% COD and 99% turbidity removal [4]. However, most studies focus primarily on general water quality indicators such as COD, BOD, or turbidity, while the concentration of surfactants, particularly cationic compounds, is rarely measured. This significantly limits understanding of their behaviour during treatment and their potential environmental impact.

Despite advancements in treatment technologies, effluent quality is often inadequate for reuse in laundry operations due to the presence of oils, surfactants, suspended solids, and high COD, as well as trace organic solvents and heavy metals [19]. Furthermore, most studies address the treatment of combined effluent from the entire wash cycle, which is typically highly polluted. As an alternative, wastewater generated during the final rinse and fabric softening stages may offer a less contaminated and more manageable stream for targeted treatment.

The number of papers related to fabric softening in the laundry process is very limited. Furthermore, despite its significant production and utilisation, only a few papers have raised the issue of esterquat removal from aqueous solutions and environmental pollution [9]. Previous research [29] proved that the application of ceramic microfiltration modules allowed capture of 70% esterquat from a model solution of a very wide range of concentrations (50–1000 mg/L).

The objectives of this study included the identification and characterization of laundry wastewater containing esterquat surfactant, based on which purification systems were implemented. The quality of treated wastewater was compared to the Polish standard for the discharge of wastewater into the environment (water and ground) [30]. The possibility of reusing recovered water was also discussed.

## 2. Materials and Methods

### 2.1. Wastewater

The laundry wastewater was obtained from the cotton fabric softening cycle in a hotel laundry located in Poland and collected in an equalization tank. The fabric softening process was carried out using a detergent containing cationic surfactant (esterquat distearoylethyl/dipalmitoylethyl dimonium chloride), benzisothiazolinone, parfum, benzyl salicylate, citronellol, hexyl cinnamal, limonene, linalool.

#### Characterization Methods

The concentration of cationic surfactants (CSs) in wastewater was measured using cuvette tests LCK331 (Hach, Wrocław, Poland), together with a DR3900 spectrophotometer (Hach, Loveland, CO, USA). Total organic carbon (TOC) concentrations were measured by means of the high temperature catalytic combustion method (550 TOC-TN, Hach, Loveland, CO, USA). The 2100N IS (Hach, USA) was used for turbidity analyses. For pH and conductivity measurements, the following devices were used: pH meter CP-315M, Elmetron (Zabrze, Poland), and conductivity meter CC-411, Elmetron (Zabrze, Poland).

### 2.2. Set-Up

**Figure 2:**
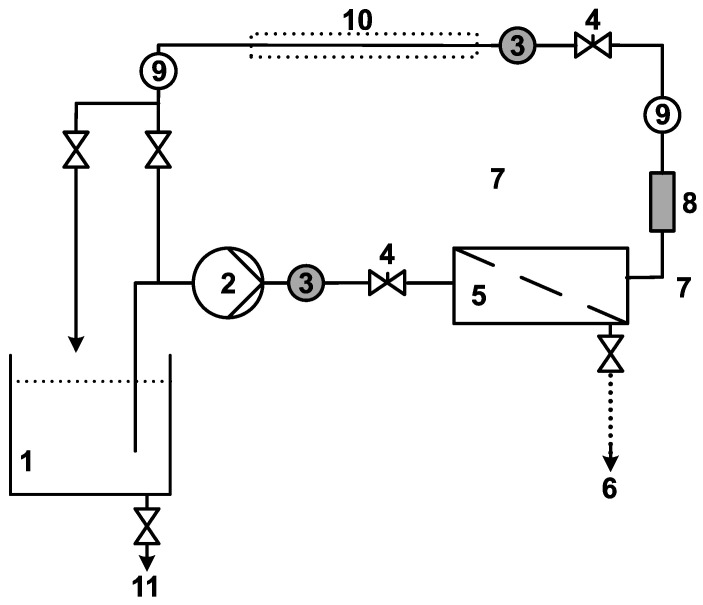
Schematic diagram of the membrane installation: (1) feeding tank, (2) pump, (3) manometer, (4) pressure regulation valve, (5) membrane module, (6) permeate, (7) concentrate, (8) rotameter, (9) thermometer, (10) cooler, (11) drain valve.

Laundry wastewater at a temperature of 22 °C was submitted for purification within 24 h after its generation. Before membrane filtration, wastewater was pretreated with bag filters (ChemTech, Wrocław, Poland) made of nylon monofilament mesh with a pore size of 100 µm. Membrane processes were carried out using the pilot installation (Figure 2).

All membrane filtration processes in this study were performed in cross-flow mode under a transmembrane pressure of 0.3 MPa. The flow velocity along the membrane surface was set at 3 m/s for MF and UF, and 0.6 m/s for NF. The system included a cooler to maintain constant temperature (22 ± 1 °C) during operation.

### 2.3. Membrane Modules

The study used microfiltration (MF) and ultrafiltration (UF) modules, as well as nanofiltration (NF) modules, the characteristics of which are presented in Table 3. Ceramic MF and UF modules were applied in the first stages of the treatment, while a denser, polymeric NF module was applied in the second stage. Ceramic membranes were chosen for their high chemical and thermal stability, low fouling tendency, and superior permeability, characteristics which should be highlighted in terms of LWW treatment in situ [21].

During the experiments, the hydraulic capacity as permeate volume flux (J) of the modules was assessed:

(1) $$J=\frac{V}{t\cdot A},\frac{L}{m^{2}h}$$

where *V* is the volume of permeate sample collected, L; *t* is time, h; and *A* is the effective area of a module surface, m^2^.

To designate the membrane’s susceptibility to fouling, the relative flux (*RF*) was evaluated:

(2) $$RF=\frac{J}{J_{0}},$$

where *J* is the permeate volume flux, L/m^2^h; and *J*_0_ is the distilled water flux, L/m^2^h.

The concentration factor (*CF*) was evaluated using the following formula:

(3) $$CF=\frac{V_{0}}{V_{t}(t)},$$

where *V*_0_ is the initial feed volume of LWW, L; *V_t_* is the feed volume of LWW at time t, L.

### 2.4. Purification Systems

Permeate collected in low-pressure-driven membrane processes (MF or UF) was submitted for the second stage of purification, which was the final membrane treatment (NF) or adsorption process (performed in a flow reactor and batch reactor). Four integrated purification systems were verified, as detailed in Table 4.

The adsorption stage was conducted using granular activated carbon (GAC) and powdered activated carbon (PAC), both supplied by Chemviron (Feluy, Belgium). GAC was used in a flow-through column mode, with a bed height of 0.15 m and a filtration rate of 5 m/h (bed volume 0.4 L). PAC adsorption was carried out in batch reactors with doses of 0.5 g/L and 1 g/L, under mixing conditions of 150 rpm for 30 min, followed by 60 min of sedimentation. After sedimentation, treated samples were filtered using 0.45 µm cellulose syringe filters (Qpore, Gdańsk, Poland) to remove residual carbon particles.

## 3. Results and Discussion

### 3.1. Wastewater Characteristics

Laundry wastewater was generated during the cotton fabric softening cycle, which included rinsing with a fabric softener fluid containing esterquat group cationic surfactants and centrifugation. The characteristics of laundry wastewater are summarised in Table 5. The concentration of CSs in the wastewater was approximately 30 mg/L, which is within the range reported in the literature (from 10 to 100 mg/L) [33]. The wastewater also exhibited significant turbidity (around 45 NTU), grey colour, and visible solid fractions, which comprised remnants of fabrics. Additionally, the samples emitted a strong odour associated with the presence of fragrances in the applied detergent.

### 3.2. Filtration

Preliminary treatment in all variants included a filtration process using bag filters with a pore size of 100 µm. As a result of filtration, the concentrations of CSs, COD, TOC, and turbidity were reduced to 21.3 mg/L, 78 mg/L, 41.6 mg/L, and 31.5 NTU, respectively, indicating an efficiency of approximately 20–30%. Solid fractions were removed.

### 3.3. Microfiltration—0.45 µm Module

In three of the proposed variants, microfiltration using a ceramic module with a pore size of 0.45 µm was applied as the primary membrane process. The permeate obtained from the MF was characterised by the following parameters: CSs 1.45 mg/L, turbidity 0.4 NTU, and TOC 16.9 mg/L, resulting in a process efficiency to decrease these factors of 93%, 98%, and 60%, respectively. Both CSs and TOC concentrations in the wastewater after MF were lower than the permissible values established by the Polish regulations [30] (5 mg CSs/L and 30 mg TOC/L). In addition, MF permeate fulfils the requirements for landscape irrigation and toilet flushing, but cannot be reused in the laundry process due to the elevated hardness. In the permeate obtained, an intense detergent odour was noticeable, leading to the decision to implement further treatment.

### 3.4. Variant I: Filtration—Microfiltration—Nanofiltration

**Figure 3:**
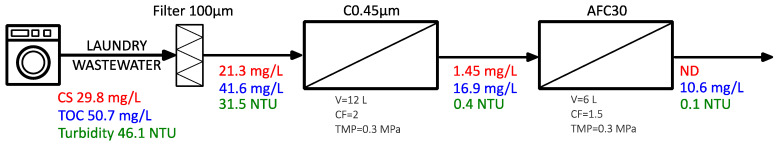
Sequential LWW treatment system: F–MF–NF.

The treatment system (Figure 3) included filtration, microfiltration using a ceramic module with a pore size of 0.45 µm, and nanofiltration using the AFC30 polymer module (to obtain 2 L of solution (concentration factor equal to 1.5)).

Membrane filtration with the AFC30 module ensured the complete removal of cationic surfactants. However, TOC removal was not complete (with the permeate containing 10.6 mg TOC/L), and was most likely due to the presence of fragrance compounds with low molecular weight. The COD of the treated wastewater was below the detection limit of the applied method.

The nanofiltration process allows for the removal of chemical compounds responsible for the hardness of the water, which, in the case of reuse of water for laundry purposes, brings many benefits, such as reduced detergent consumption, improved washing efficiency, and extended lifespan of industrial laundry equipment.

The wastewater after MF directed to the nanofiltration module had a total hardness of 15.1° dH (268.8 mg CaCO_3_/L), indicating a medium hardness. The use of the AFC30 module reduced the hardness to 4.1° dH (73 mg CaCO_3_/L), resulting in very soft water that meets the hardness standard for laundry water [18].

**Figure 4:**
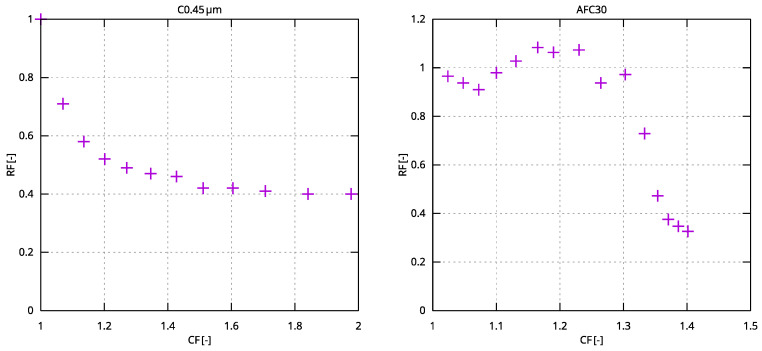
Relative permeability of MF (C0.45 µm) and NF (AFC30) modules vs. concentration factor (TMP = 0.3 MPa).

**Figure 5:**
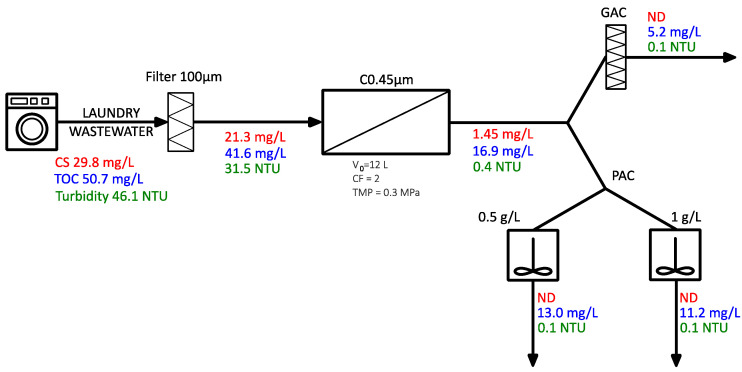
Sequential LWW treatment system: F–MF–ADS.

LWW treatment was associated with a decrease in hydraulic performance of the MF module to approximately 0.4 (Figure 4). It is important to note that the most significant decrease in relative flux values occurred during the initial stages of filtration, and after reaching a concentration factor (CF) of 1.5, no further deterioration in the module’s relative permeability was observed. The removal of a substantial portion of organic contaminants by the microfiltration module effectively mitigated fouling in the second stage of membrane treatment (Figure 5). At the beginning of the process, the relative permeability of the AFC30 module dropped to 0.8 (at a CF of approximately 1.2), after which the module’s permeability subsequently improved, resulting in a flux slightly higher than that of distilled water. A decrease in permeate flux was related to the interaction of the surfactant micelles with the membrane surface and pore structure. In particular, cationic surfactants, such as esterquats, are known to adsorb onto negatively charged membrane surfaces due to electrostatic and hydrophobic interactions. These mechanisms, along with micelle-induced gel layer formation, have been described in detail in our previous works [29,34], where the influence of surfactant type and concentration on flux decline and pore blockage was systematically investigated.

### 3.5. Variant II, III: Filtration—Microfiltration—Adsorption

Adsorption using activated carbon is a widely studied method for the removal of organic pollutants, including surfactants, from effluents [35,36,37,38]. This method was shown to be effective for the removal of fragrances and odours [39,40,41]. The No. II and III designed systems (Figure 5) involved filtration, microfiltration, and adsorption on activated carbon, implemented in three variants. In the first variant, the permeate obtained from the MF was directed to a GAC column, where the adsorption was carried out under flow conditions. In the second and third systems, powdered activated carbon was used for the adsorption process at doses of 0.5 and 1 g/L, respectively. The experiments were carried out in batch reactors with rapid mixing (150 rpm) for 30 min, followed by 60 min of sedimentation.

The implementation of activated carbon adsorption as the final stage of treatment resulted in lower concentrations of total organic carbon (TOC) compared to the sequence of F–MF–NF processes. In the flow system, the TOC concentration in the averaged sample decreased to approximately 5 mg/L, while under batch conditions it decreased to approximately 13 mg/L and 11 mg/L at carbon doses of 0.5 and 1 g/L, respectively. The higher adsorption efficiency observed in the flow system is likely due to the larger mass of the adsorbent relative to the mass of the adsorbate. In all variants tested, the treated solution was odourless.

### 3.6. Variant IV: Filtration—Ultrafiltration—Nanofiltration

**Figure 6:**
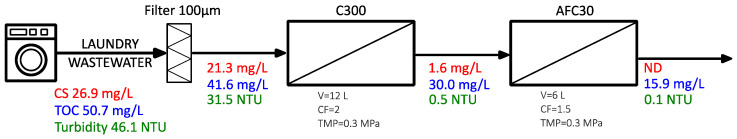
Sequential LWW treatment system: F–UF–NF.

In the fourth treatment system (Figure 6), ultrafiltration (C300) and nanofiltration (AFC30) were applied after the filtration process. Membrane processes were carried out at a transmembrane pressure (TMP) of 0.3 MPa, achieving a concentration factor of 2 for the UF module and 1.4 for the NF module, respectively.

**Figure 7:**
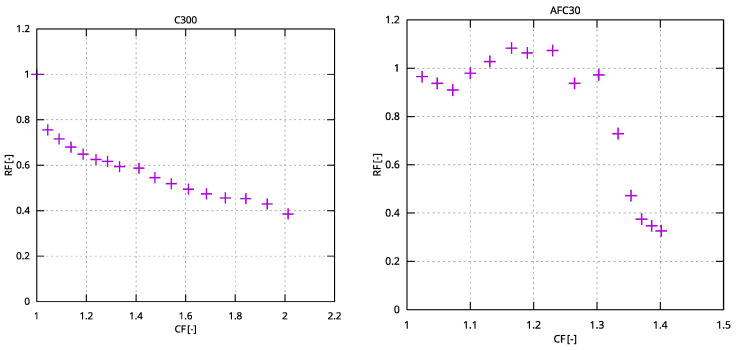
Relative permeability of UF (C300) and NF (AFC30) modules vs. concentration factor (TMP = 0.3 MPa).

LWW treatment led to a decrease in the relative permeability of the C300 module to approximately 0.4 at the end of the experiment, corresponding to a flux of approximately 40 L/m^2^h. The study demonstrated that the UF module exhibited a similar susceptibility to fouling (Figure 7) as the microfiltration module C0.45 µm (Figure 5), but with a lower separation efficiency for contaminants. The TOC concentration in the UF permeate was approximately twice as high as in the MF permeate, resulting in an increased organic load entering the AFC30 module in the next treatment stage.

Using the AFC30 polymer nanofiltration module in the next step of purification resulted in a reduction of the concentration of cationic surfactants below the detection limit of the applied analytical method, and a reduction of TOC by approximately 50%. However, compared to the F–MF–NF system, there was a noticeable deterioration in the hydraulic performance of the AFC30 module (Figure 7), likely due to the higher concentration of organic contaminants entering the module. The wastewater after the sequence of the treatment process had a characteristic detergent odour.

The relationship between membrane pore size and process performance plays a key role in the design of membrane-based treatment systems. In general, membranes with smaller pore sizes, such as nanofiltration, offer higher separation efficiency for organic compounds and surfactants. However, they typically operate at lower permeate fluxes and require higher transmembrane pressures, increasing pumping energy demand and operational costs. To address these limitations and make optimal use of each membrane stage, a sequential treatment approach was proposed. By combining different membrane processes and adsorption stages, the system was designed to maximize pollutant removal while minimizing the negative effects associated with pressure loss and fouling intensity. The treatment efficiency obtained in this study, including complete removal of cationic surfactants and high reductions in turbidity and total organic carbon, is consistent with or higher than the results reported in previous studies [16,20]. Our findings confirm the effectiveness of combining adsorption and membrane filtration for the treatment of final rinse wastewater containing cationic surfactants; thus, they fill the gap in this research area.

## 4. Conclusions

The study demonstrated the effectiveness of integrated membrane-based processes combined with conventional purification techniques for treating laundry wastewater obtained in the softening stage of cotton fabrics. The wastewater treated in the filtration–microfiltration and filtration–ultrafiltration processes met the regulatory standards for the discharge of industrial wastewater and could be reused for landscape irrigation and flushing of toilets. Furthermore, the implementation of nanofiltration as a final step produced a filtrate suitable for reuse in the laundry process because of the removal of hardness. Activated carbon adsorption proved to be a promising post-treatment step, significantly reducing organic compound concentrations and eliminating odours. The choice of treatment method should be based on the intended reuse of the recovered water.

Despite these promising results, challenges remain to minimise the membrane fouling phenomenon which is significant because of the surfactant action. Another crucial challenge is the management of concentrates generated during membrane separation processes. These streams contain high concentrations of pollutants that pose challenges in their disposal.

The study highlights the potential of integrated wastewater treatment systems to improve water recovery, minimise environmental impact, and support sustainable industrial practices.

## Figures and Tables

**Table 1:** Characteristics of LWW.

Reference	Type of LWW	pH [-]	Turbidity [NTU]	TS [mg/L]	BOD_5_ [mg O_2_/L]	COD [mg O_2_/L]	Surfactants [mg/L]
[5]	Domestic	7.46	437	N/A	N/A	286	45
[13]	Domestic	9.58	9.76	N/A	1546	3135	38.9
[14]	Domestic	9.66 ± 0.03	45 ± 1.42	2311.83 ± 11.77	N/A	N/A	40.82 ± 0.1 (anionic)
[15]	Hospital	6.6–11.7	37 * ± 29	363 ± 146	N/A	411 ± 204	N/A
[16]	Industrial	10.0 ± 0.1	61 ± 2	456 ± 6	58 ± 0	587 ± 4	11.7 ± 0.1
		10.5 ± 0	52 ± 2	530 ± 3	87 ± 0	383 ± 15	19.6 ± 0.1
		10.9 ± 0	64 ± 1	532 ± 7	67 ± 0	245 ±8	15.9 ± 0
[17]	Industrial	11.08 ± 1.30	1290 ± 105	2900 ± 326	750 ± 80	1920 ± 220	9.42 ± 0.85 (LAS) 37.36 ± 0.17 (BiAS)
[4]	Industrial	7–9	40–150	90–200	N/A	400–1000	1–10 (nonionic) 1–15 (anionic)
[18]	Domestic	9.3–10	14–400	200–987	48–1200	375–4155	N/A
	Industrial	9.0–11	40–150	400–1000	218–9810	80–212,000	N/A
	Hospital	11.4–11.6	87.9	66–71	44–50	477–876	N/A

* value as FAU; N/A–not available.

**Table 2:** Water reuse standards established to ensure environmental protection, human health safety, and compatibility with the intended application [18].

	Landscape Irrigation	Toilet Flushing	Laundry Process
pH [-]	6.0–9.0	6.0–9.0	6.0–9.0
Turbidity [NTU]	no limit	no limit	2
BOD_5_ [mg O_2_/L]	30	30	10
TSS [mg/L]	30	30	10
Hardness [mg CaCO_3_/L]	no limit	no limit	90

**Table 3:** Module characteristics.

Symbol	Material	Cut-Off [kDa]	Pore Size [nm]	Distilled Water Flux * [L/m^2^h]
MF C0.45 µm	zirconium dioxide	-	450	240
UF C300	zirconium dioxide	300	-	101
NF AFC40	polyamide	0.2 [31]	0.51 ± 0.10 [32]	16

* Determined by the authors (*TMP* = 0.3 MPa, *T* = 22 °C).

**Table 4:** Integrated purification systems with process parameters.

Variant	Stage I		Stage II	
I F–MF–NF	Microfiltration		Nanofiltration	
	MF C0.45 µm	*V*_0_ = 10 L	NF AFC30	*V*_0_ = 5 L
		*TMP* = 0.3 MPa		*TMP* = 0.3 MPa
		*ν* = 3 m/s		*ν* = 0.6 m/s
		*CF* = 2		*CF* = 1.5
II F–MF–ADS	Microfiltration		Adsorption, flow reactor	
	MF C0.45 µm	*V*_0_ = 10 L	GAC	*V*_0_ = 3 L
		*TMP* = 0.3 MPa		*H* = 0.15 m
		*ν* = 3 m/s		*V_B_* = 0.4 L
		*CF* = 2		*v* = 5 m/h
III F–MF–ADS	Microfiltration		Adsorption, batch reactor	
	MF C0.45 µm	*V*_0_ = 10 L	PAC	*V*_0_ = 1 L
		*TMP* = 0.3 MPa		*D_C_* = 0.5; 1 g/L
		*ν* = 3 m/s		*t_m_* = 30 min
		*CF* = 2		*t_s_* = 60 min
IV F–UF–NF	Ultrafiltration		Nanofiltration	
	UF C300	*V*_0_ = 10 L	NF AFC30	*V*_0_ = 5 L
		*TMP* = 0.3 MPa		*TMP* = 0.3 MPa
		*ν* = 3 m/s		*ν* = 0.6 m/s
		*CF* = 2		*CF* = 1.5

**Table 5:** Laundry wastewater characteristics.

Parameter	Wastewater	Standard for Discharge into the Environment [30]
pH [-]	7.16 ± 0.30	6.5–9
Conductivity [µS/cm]	773.5 ± 54.1	-
Turbidity [NTU]	46.1 ± 1.9	-
CSs [mg/L]	29.8 ± 7.9	5 *
COD [mg O_2_/L]	86.4 ± 12.3	125
BOD_5_ [mg O_2_/L]	74.0 ± 5.7	25
TOC [mg/L]	50.7 ± 11.3	30
Total solids [mg/L]	343 ± 7	35

* Polish legislation provides limits only for anionic and non-ionic surfactants. Due to the significant toxicity of cationic surfactants, the strictest value, i.e., the limit for anionic surfactants (5 mg/L), has been adopted for the purposes of this study.

## Data Availability

The original contributions presented in this study are included in the article. Further inquiries can be directed to the corresponding authors.

## Abbreviations

The following abbreviations are used in this manuscript:

A	Effective Membrane Surface Area (m^2^)
BOD_5_	Biochemical Oxygen Demand (5-day)
CF	Concentration Factor (–)
COD	Chemical Oxygen Demand
CSs	Cationic Surfactants
DC	Dose of Carbon (g/L)
F	Filtration
GAC	Granular Activated Carbon
H	Bed Height (m)
J	Permeate Volume Flux (L/m^2^·h)
J_0_	Distilled Water Flux (L/m^2^·h)
LWW	Laundry Wastewater
MF	Microfiltration
NF	Nanofiltration
NTU	Nephelometric Turbidity Unit
PAC	Powdered Activated Carbon
RF	Relative Flux (J/J_0_)
t	Time (h or min, depending on context)
t_m_	Mixing Time (min)
t_s_	Sedimentation Time (min)
TMP	Transmembrane Pressure (MPa)
TOC	Total Organic Carbon
TS	Total Solids
TSS	Total Suspended Solids
UF	Ultrafiltration
v	Filtration Rate
VB	Volume of Carbon Bed (L)
V_0_	Initial Feed Volume (L)
V_t_	Volume at Time t (L)
ν	Flow velocity along membrane surface (m/s)
